# Non-invasive measurement of blood pressure and its possible use in future in-depth studies on farrowing-related complications

**DOI:** 10.1186/s40813-026-00512-6

**Published:** 2026-04-21

**Authors:** Anna Carlertz, Magdalena Jacobson, Patricia Hedenqvist, Anneli Rydén

**Affiliations:** 1 Farm & Animal Health, Skara, SE-532 89 Sweden; 2 Department of Clinical Science, Swedish University of Agricultural Sciences, PO Box 7054, Uppsala, SE-75007 Sweden

**Keywords:** Blood pressure, Non-invasive, Sow, Gestation, Farrowing, Systolic, Diastolic

## Abstract

**Background:**

Farrowing is the most critical phase in a sow’s production cycle and has major implications for both animal welfare and productivity. Early identification of sows at risk of farrowing-related disorders is therefore essential. In human obstetrics, blood-pressure measurement is a key tool for detecting individuals at increased risk of pregnancy-related complications, and both hypertension and excessive increase in blood pressure have been associated with adverse outcomes. Previous work has suggested that low systolic blood pressure may be linked to prolonged farrowing, a condition known to increase stillbirth rates and neonatal mortality. Invasive arterial catheterisation remains the reference method for blood-pressure assessment but is impractical for routine or repeated use in pregnant sows, whereas non-invasive oscillometric tail-cuff techniques have shown potential feasibility. The aim of this study was to evaluate a non-invasive method for blood-pressure monitoring in sows and to describe preliminary gestational blood-pressure patterns that may inform future work on identifying individuals at risk of farrowing-related complications.

**Results:**

Measurements could be performed as planned during all stages of gestation and lactation. The non-invasive tail-cuff method functioned well both when the sows were confined in feeding stalls and when they were moving freely in the farrowing pens. Non-invasive blood-pressure measurements revealed a progressive pattern of decline in systolic, diastolic and mean blood pressures throughout the sows’ gestation.

**Conclusion:**

Non-invasive blood-pressure measurement in sows under commercial production conditions proved feasible, and the measurements obtained in this study demonstrated a significant decline in blood pressure during late gestation. These findings highlight the need for further research to establish reliable reference intervals and to determine thresholds for critically low blood pressure in relation to farrowing.

## Background

Farrowing represents the most critical period in a sow’s life cycle and has a major impact on production outcomes [1, 2]. Early identification of sows at risk of developing health disorders during farrowing is therefore essential for optimising reproductive performance and animal welfare [3, 4]. Nevertheless, substantial knowledge gaps remain regarding the physiological mechanisms underlying various complications during gestation and farrowing [5].

In pregnant women, blood-pressure measurement is an important tool for identifying individuals at increased risk of pregnancy-related complications [6]. It has been demonstrated that not only hypertension, but also an excessive rise in blood pressure during pregnancy, is associated with adverse outcomes [7]. These findings have raised the question of whether similar patterns can be observed in pregnant sows.

As early as 1993, Madec and Léon conducted a study attempting to measure blood pressure in late-gestation sows to examine potential associations between blood pressure and farrowing duration, and they reported that low systolic pressure was associated with prolonged farrowing [8]. In a more recent study, Schoos et al. reported that prolonged farrowing is associated with increased stillbirth rates and neonatal mortality, emphasising the importance of identifying sows at risk [9]. To measure blood pressure, invasive arterial catheterisation remains the reference standard [10], but it is time-consuming, technically demanding, and ethically unsuitable for repeated measurements in pregnant sows. Non-invasive methods, such as oscillometric tail-cuff measurements, have been reported to be feasible in pigs [8].

In larger litters, the uterine blood flow is dispensed among more foetuses, which reduces the amount of nutrients and oxygen that each individual will achieve. This may partly explain why piglets from large litters tend to have lower birth weights [11]. In sows, blood and plasma volume increase during pregnancy and up to 14–21 days into lactation, after which it decreases again, but there is no correlation between plasma volume and litter weight [12]. Madec and Léon observed a correlation between low systolic blood pressure and prolonged farrowing duration, which suggests that blood-pressure measurement could be a method to identify at-risk sows before farrowing [8]. To apply blood-pressure measurement effectively in practice, a simple and repeatable technique is required, along with reference values for the method.

The aim of this study was to evaluate a non-invasive method for blood-pressure monitoring in sows and to describe preliminary gestational blood-pressure patterns that may inform future work on identifying individuals at risk of farrowing-related complications.

## Materials and methods

The study was conducted at the Swedish Livestock Research Centre, Funbo-Lövsta, Uppsala, Sweden, between June and October 2022. Ethical approval was obtained from the Uppsala Ethics Committee on Animal Research (permit number Dnr 5.8.18–09060/2022). All procedures were conducted in accordance with EU Directive 2010/63/EU on the protection of animals used for scientific purposes.

The experimental herd at the Swedish University of Agricultural Sciences (SLU) research farm in Funbo Lövsta is an SPF-herd that houses approximately 132 sows. Six breeds or breed combinations were represented: Y (purebred Swedish Yorkshire), LY (Landrace × Yorkshire), LZ (Landrace × Canadian Yorkshire, the “Z-line”), ZY, ZYY (alternating backcross; 25% Z-line and 75% Swedish Yorkshire), and ZZY. Gestating sows were kept on deep straw-litter bedding in stable groups of 8–12 animals. During gestation, they were fed twice daily in individual feeding stalls, where they were temporarily confined to prevent competition during feeding. The herd operates a two-week batch-farrowing system, and all sows farrow in free-farrowing pens. The average gestation length in the herd was 116 ± 3 days. A 16-hour light programme was maintained throughout the study period. The sow parity ranged from 1 to 8, body weight ranged from approximately 250 to 350 kg, and all animals remained clinically healthy throughout the experiment.

The initial blood-pressure (BP) measurements were performed approximately 30 min after feeding, while the sows were confined in the feeding stalls. The measurements were carried out on four groups of sows representing different reproductive stages: nine sows in early gestation (days 20–30), nine sows in mid-gestation (day 60), seven sows in late gestation (day 115), and ten lactating sows (10–14 days post-partum). Sows ranged from parity 2 to 9, with a median of 4. Measurements were conducted after the morning feeding between 08:00 and 11:00.

After the initial measurements, six of the sows first examined during early gestation (days 20–30) were followed longitudinally with repeated measurements at days 94–104 and at days 108–114. Measurements at day 108–114 were performed in the free-farrowing pens without confining the animals.

**Fig. 1:**
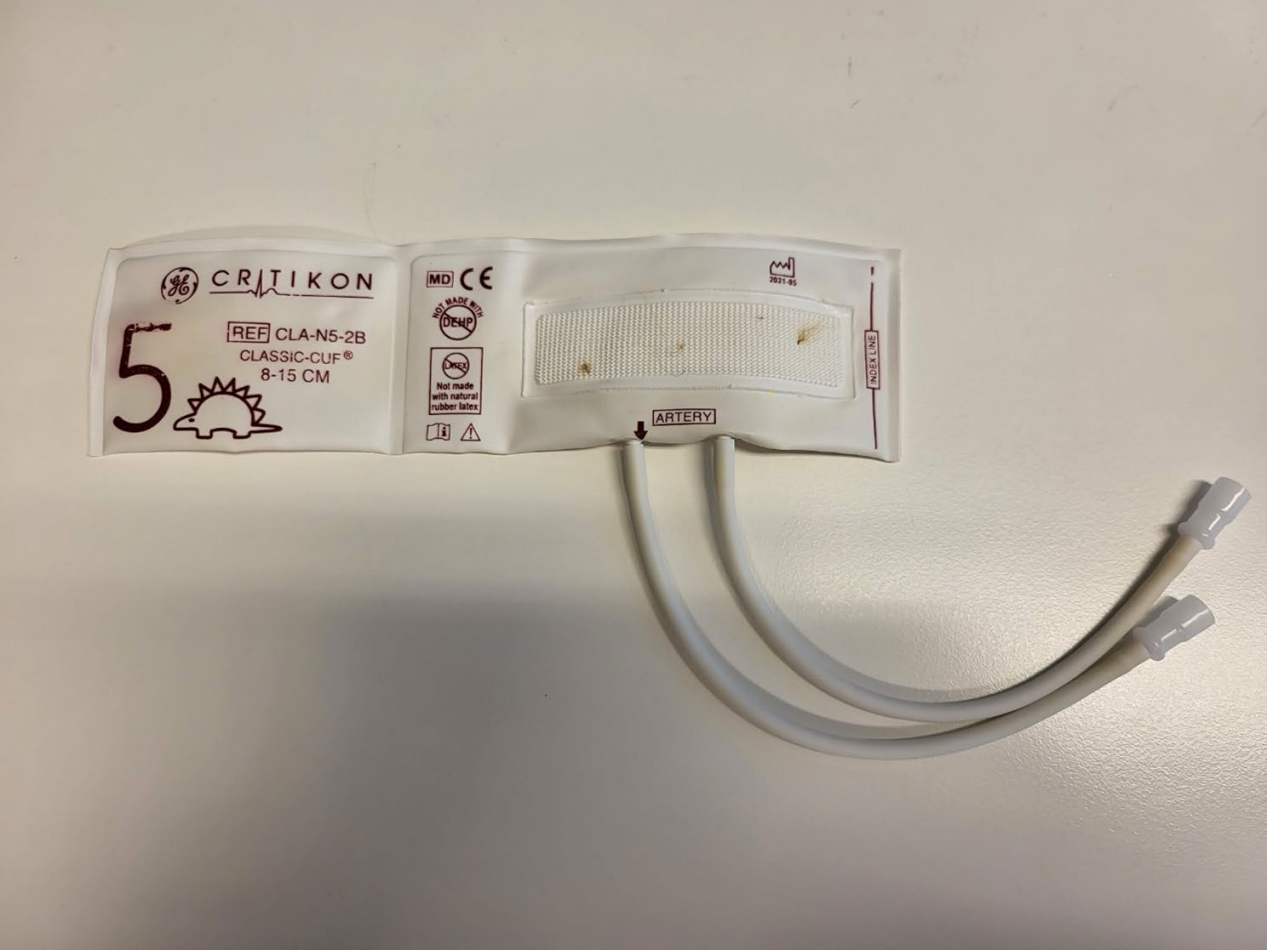
Adjustable blood-pressure cuff used for tail measurements in sows, with a band circumference of 8–15 cm

**Fig. 2:**
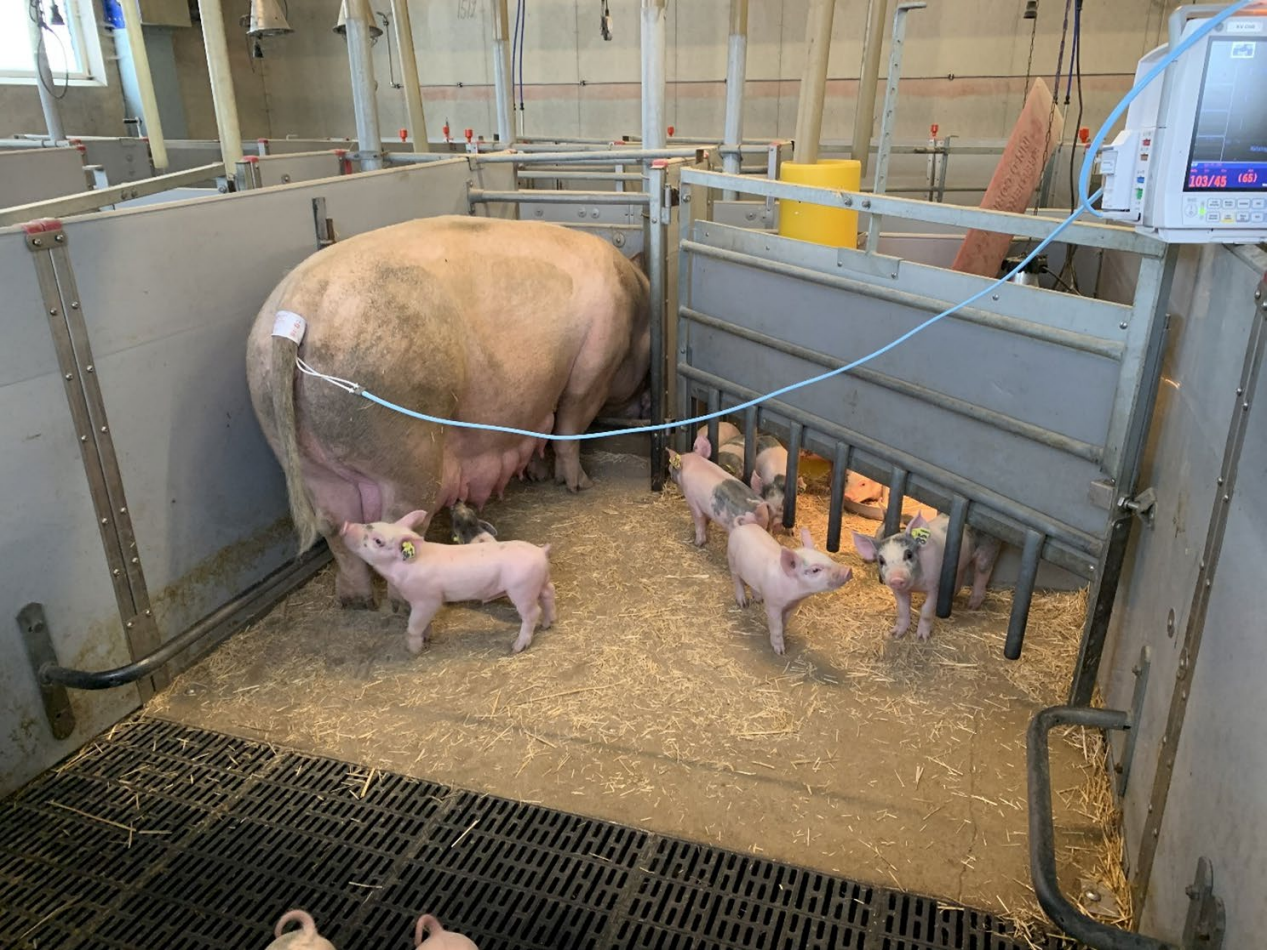
Illustrative photograph showing placement of the blood-pressure cuff at the base of the tail over the coccygeal artery in a free-moving sow in a farrowing pen

Non-invasive blood-pressure measurement was carried out using a GE B40 Patient Vitals Monitor (GE Healthcare, USA) equipped with a Critikon Neonate 5 Pet Cuff for Animal Blood-Pressure Measurement Cuff PVC (GE Healthcare, USA). The cuff was designed for veterinary use, with a circumference range of 8–15 cm and a width of less than 5 cm (Fig. 1). It featured a 3.6 m cable, which allowed the monitor to be positioned outside the farrowing pen to minimise disturbance. The cuff was placed at the base of the tail, corresponding to the location of the coccygeal artery (Fig. 2). All sows had intact tails.

For each sow, three to five consecutive readings were taken at one-minute intervals. The mean value used for analysis was calculated from three to five consistent measurements obtained during each session. The mean of at least three consistent measurements was used for analysis. The sow’s body posture (standing, sitting, or lying) was recorded, and the vertical distance between the tail base and heart level was measured for hydrostatic correction. Because cuff height relative to heart level changes with posture, all measurements were hydrostatically corrected. To enable this correction, the vertical distance from the sow’s heart level to the floor and the distance from the cuff to the floor were measured at the time of each recording. Heart level was approximated anatomically as the level of the thoracic cavity just behind the elbow. To increase accuracy, the heart region was confirmed by auscultation before the vertical distance from heart level to the floor was measured. The difference between these two measurements (Δh, in cm) was inserted into the hydrostatic correction formula:

$$\:{\mathrm{BP}}_{\mathrm{corrected}}={\mathrm{BP}}_{\mathrm{measured}}+(0.74\times\:{\Delta\:}h)$$

where Δh is positive when the cuff is positioned below the heart level and negative when the cuff is above the heart level. The correction factor of 0.74 mmHg/cm corresponds to the hydrostatic pressure difference due to blood density. It is widely used to adjust non-invasive blood-pressure measurements to heart level [13] and was applied to all blood-pressure values.

Measurements could be performed as planned during all stages of gestation and lactation. The non-invasive tail-cuff method functioned well both when the sows were confined in feeding stalls and when they were moving freely in the farrowing pens.

Of the nine sows initially included in the early-gestation group (i.e. days 20–30), six remained in the herd until the time of farrowing. Three sows were excluded during the study period because they had returned to oestrus or had been culled during gestation.

Most sows tolerated the blood-pressure measurements well and showed no obvious signs of stress or discomfort (Fig. 2). A few sows moved slightly forward in the feeding stall during the first measurement but no longer reacted during subsequent sessions. In some cases, minor tail movements or attempts to withdraw the tail were observed when the cuff was first applied, but such reactions usually ended within a few seconds.

It was possible to obtain three to five consecutive readings within a few minutes without disturbing the animals. The quality of the measurements was considered good because consecutive readings within each measurement session showed only minor variation. The equipment produced stable and repeatable values in most cases, allowing mean values to be calculated for all time points.

### Statistical analysis

All statistical analyses were conducted using InVivoStat Graphics version 4.3 (2021); InVivoStat, UK.

Data were examined for normality using diagnostic plots. Blood-pressure data from the four groups of sows representing different reproductive stages were rank transformed and compared using one-way ANCOVA while controlling for parity, with all pairwise comparisons without adjustment for multiplicity (LSD test). The data from the six sows followed longitudinally across their pregnancies were normally distributed and analyzed using repeated measures mixed model approach, with treatment factor Day-of-pregnancy. This was followed by planned comparisons on the predicted means to compare the levels of the Day-of-pregnancy.

## Results

### Measurements on sows in different reproductive stages

**Fig. 3:**
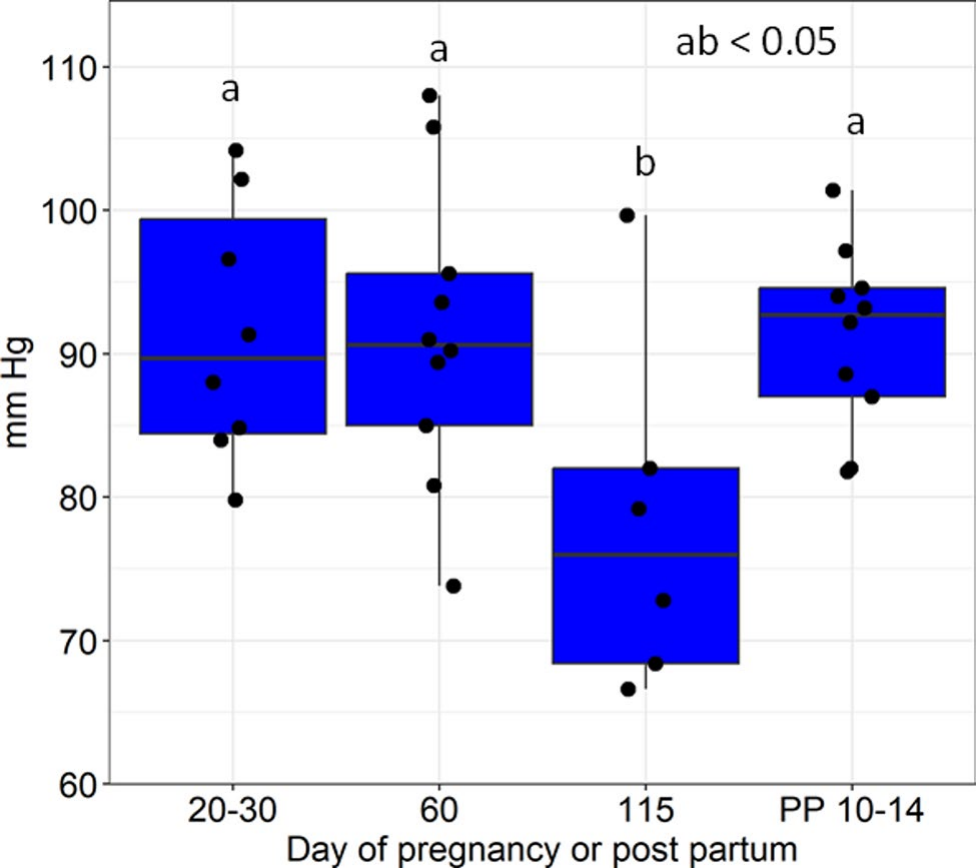
Box plot of mean blood pressure in independent sow groups at each stage of gestation and lactation. ab, *p* < 0.05 compared to all other time points. ANCOVA on rank transformed data and parity as a covariate followed by all pair-wise comparisons without adjustment for multiplicity (LSD test). PP=postpartum day. There was no effect of parity (*p* = 0.2)

There was no overall difference for level of Day (*p* = 0.056) whereas pairwise comparisons showed that mean blood pressure was lower at 115 days of pregnancy than at all other time points (Fig. 3.). Parity had no significant effect on mean blood pressure (*p* = 0.2). Systolic blood pressure was likewise lower at 115 days compared to all other time points (115 d *vs. post-partum* day 10–14, *p* < 0.01; 115 d vs. day 20–30, *p* < 0.05 and 115 d vs. day 60, *p* < 0.05. Mann-Whitney tests). Diastolic blood pressure was only lower at 115 days as compared to days 20–30 (*p* < 0.05, Mann-Whitney test).

### Repeated measures in 6 sows

**Fig. 4:**
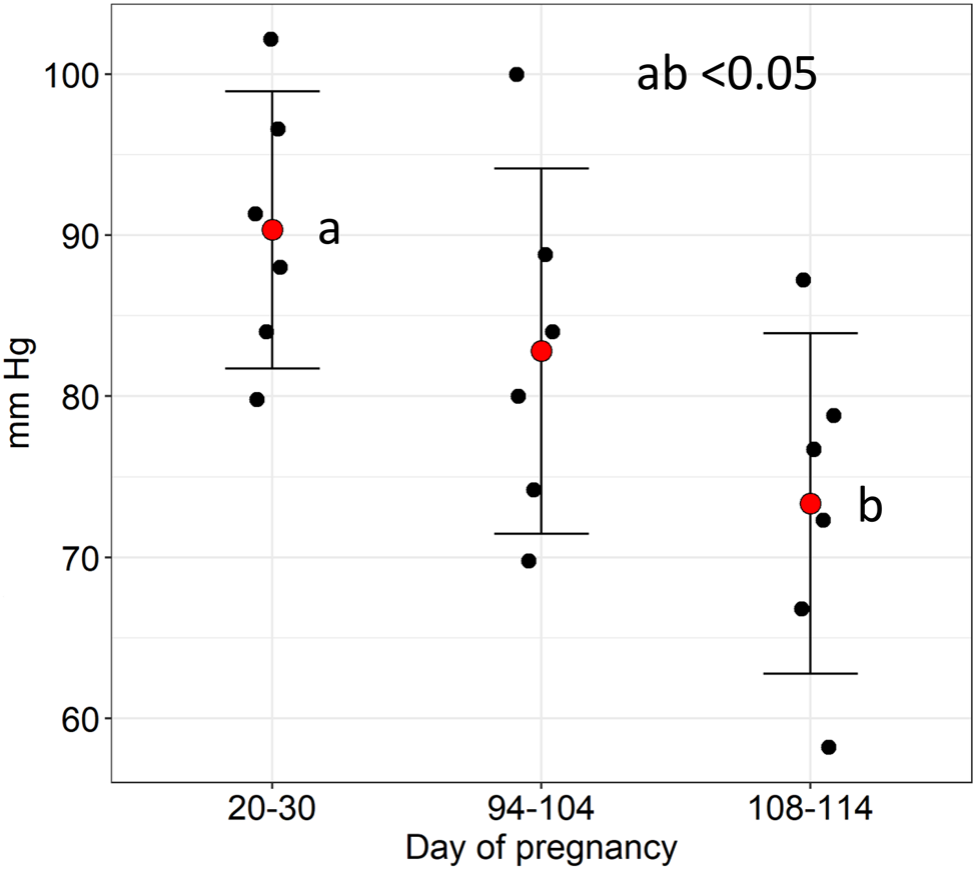
Difference in mean blood-pressure within the same individuals during day 20–23, 94–104, and 108–114. Data presented as medians, confidence intervals and individual data. ab, *p* < 0.05. Repeated measures mixed model

The repeated-measures mixed model identified a significant overall difference in mean BP across the days of pregnancy (F=4.24, *p*=0.046). There was a decrease in the blood pressure from day 20-30 to day 94-104 (difference = –16.98, 95% CI: –29.97 to –3.98, *p* = 0.015) (Fig 4.) No significant differences were detected between days 20-30 and pregnancy days 94-104 (*p* = 0.23), nor between pregnancy days 94-104 and late pregnancy (*p* = 0.14). Profile plots demonstrated a consistent pattern across all six sows, with individual trajectories showing a decline from early toward late gestation.

There was no difference between the days of pregnancy for systolic or diastolic BP (non-normal distribution, Wilcoxon Signed Rank tests).

## Discussion

Invasive blood-pressure measurement is regarded as the gold standard for accuracy in both human and veterinary medicine. However, it is not a feasible method in clinical practise, as it requires catheterisation in anaesthetised animals and involves both technical and animal welfare limitations [8]. The results from the present study suggests that the non-invasive oscillometric tail-cuff method used provides reliable estimates of systolic and diastolic blood pressure in sows. This observation aligns with previous attempts to measure blood pressure in pigs using indirect methods [8].

Previous studies have shown that restraint and handling of pigs may lead to alterations in blood pressure [14]. However, the sows in our study tolerated the procedure well and showed minimal signs of discomfort. A few animals moved slightly forward in the feeding stall during the first session when the cuff was tightened, but such behaviour disappeared in subsequent measurements, suggesting rapid habituation [15]. Overall, the method demonstrated good practical applicability and was well tolerated by the sows, indicating that tail-cuff-based blood-pressure measurement can be successfully implemented in a clinical setting. A relevant consideration is tail status, as the extent of tail docking may influence cuff placement and may limit the applicability in herds employing this practice. In the present study, all sows had intact tails.

Measurements in the farrowing pens were somewhat more time-consuming than those performed during gestation, mainly because the sows moved freely around. The tail cuff occasionally slipped off but could easily be repositioned, and otherwise the procedure was as straightforward as in the feeding stalls. Measurements could therefore be carried out without affecting the sows’ behaviour or the care of their piglets (Fig. 2).

The ability to reliably monitor blood pressure in sows has practical implications for both welfare and production outcomes. Alterations in blood pressure may reflect physiological strain or circulatory disturbances, and early identification of deviations from normal ranges could help detect individuals at risk of complications during late gestation or farrowing [8]. A simple, repeatable and non-invasive method suitable for field conditions would therefore be a valuable tool in herd health programmes, potentially improving reproductive outcomes, reducing periparturient losses and enhancing overall sow welfare.

The results show a clear decline in blood pressure from early to late gestation, which has also been described in mares during late gestation [16]. Madec and Léon [8] also showed a correlation between low systolic pressure and prolonged farrowing. Systolic pressure and haemoglobin concentration are positively correlated in humans [17], and low haemoglobin levels are also negatively correlated with farrowing duration [18–20].

In the sows followed longitudinally, the measurement was initially planned to take place during mid-gestation; however, due to circumstances beyond our control, this measurement occurred considerably later (days 94–104).

In this study, no assessment was made on the outcome of the farrowing in relation to blood pressure, thus requiring further studies. Further research is also needed to establish reference values for what may be considered a critically low blood pressure.

## Conclusion

Non-invasive blood-pressure measurement in sows under commercial production conditions proved feasible and the measurements obtained in this study demonstrated a significant decline in blood pressure during late gestation. These findings highlight the need for further research to establish reliable reference intervals and to determine thresholds for critically low blood pressure in relation to farrowing.

## Data Availability

The datasets used and analysed during the current study are available from the corresponding author upon request.

## Abbreviations

BP: Blood pressure
